# Human Monoclonal Antibodies against Highly Conserved HR1 and HR2 Domains of the SARS-CoV Spike Protein Are More Broadly Neutralizing

**DOI:** 10.1371/journal.pone.0050366

**Published:** 2012-11-21

**Authors:** Hatem A. Elshabrawy, Melissa M. Coughlin, Susan C. Baker, Bellur S. Prabhakar

**Affiliations:** 1 Department of Microbiology and Immunology, College of Medicine, University of Illinois at Chicago, Chicago, Illinois, United States of America; 2 Centers for Disease Control and Prevention, Measles, Mumps, Rubella and Herpes Virus Laboratory Branch, Atlanta, Georgia, United States of America; 3 Department of Microbiology and Immunology, Loyola University Chicago Stritch School of Medicine, Maywood, Illinois, United States of America; Jagiellonian University, Poland

## Abstract

Immune sera from convalescent patients have been shown to be effective in the treatment of patients infected with Severe Acute Respiratory Syndrome Virus (SARS-CoV) making passive immune therapy with human monoclonal antibodies an attractive treatment strategy for SARS. Previously, using Xenomouse (Amgen British Columbia Inc), we produced a panel of neutralizing Human monoclonal antibodies (HmAbs) that could specifically bind to the ectodomain of the SARS-CoV spike (S) glycoprotein. Some of the HmAbs were S1 domain specific, while some were not. In this study, we describe non-S1 binding neutralizing HmAbs that can specifically bind to the conserved S2 domain of the S protein. However, unlike the S1 specific HmAbs, the S2 specific HmAbs can neutralize pseudotyped viruses expressing different S proteins containing receptor binding domain sequences of various clinical isolates. These data indicate that HmAbs which bind to conserved regions of the S protein are more suitable for conferring protection against a wide range of SARS-CoV variants and have implications for generating therapeutic antibodies or subunit vaccines against other enveloped viruses.

## Introduction

Severe Acute Respiratory Syndrome Coronavirus (SARS-CoV) infection in humans results in Acute Respiratory Distress Syndrome (ARDS) in 20–30% of patients with 10% mortality [1]. Passive antibody therapy has been successfully used to treat patients infected with SARS-CoV [2]–[4], and to confer protection against lethal challenge in experimental animals [5]. Re-emergence of SARS in humans remains a credible health threat because of the animal reservoirs [6]–[9]. As of now, there is no effective treatment for SARS. However, since virus titer peaks 10 days post-infection [1], [10], post-exposure treatment that is effective against a broad spectrum of viral variants remains a viable option. Many of the reported HmAbs against SARS-CoV fail to neutralize all of the clinical isolates [11]–[13]. Therefore, there is a need for a clinically usable therapy against SARS-CoV infection.

The Spike (S) glycoprotein plays an essential role in receptor binding and membrane fusion critical for the virus entry, and contains epitopes that elicit neutralizing Abs [14]–[17]. The SARS-CoV S protein consists of two functional domains, S1 (amino acids 12–680) and S2 (amino acids 681–1255) [18]. The receptor binding domain (RBD) (amino acids 318–510) contained within the S1 domain is required for binding to ACE-2 receptor on the cell surface and is thought to contain the majority of neutralizing epitopes [14], [19], [20]. Co-crystallization of the RBD and human ACE-2 identified the receptor binding motif (RBM) (amino acids 424–494) in direct contact with ACE2 [18]. The S2 domain contains the fusion peptide followed by two conserved heptad repeats (i.e. HR1 and HR2), which upon cleavage by cathepsin-L associate to form a fusion core [15], [18], [21]–[23], and facilitate fusion with the cell membrane required for the virus entry [24]. Synthetic HR2 peptides as well as HR2 specific antibodies have been shown to block SARS-CoV infection [25]–[27]. The RBD shows high rates of mutation which allows the virus to escape neutralization by Abs without losing its ability to infect cells [13], [28]. In contrast, the S2 domain is highly conserved among different clinical isolates of the SARS-CoV [29], [30], and thus raise the possibility that Abs against this region may confer better protection against a broad spectrum of clinical isolates.

Previously, using Xenomouse (mouse immunoglobulin genes were replaced by human immunoglobulin genes) immunized with SARS-CoV Urbani strain S protein ectodomain, we produced a panel of 19 neutralizing HmAbs and found that they all bound to the S1 region of the S protein [19]. We found that 18 HmAbs bound to RBD and neutralized the virus by blocking virus binding to the ACE-2 receptor, while one HmAb (4D4) neutralized the virus by inhibiting a post-binding event [11]. In this study, we describe neutralizing HmAbs that specifically bind to S2 region and found that these HmAbs, unlike S1 specific HmAbs, were better able to neutralize a broader range of surrogate clinical isolates.

## Materials and Methods

### Construction of Expression Plasmids for SARS-CoV 12-510 S1-IgG and Full Length Spike (S) Protein Mutants

The expression plasmid encoding 12-510 S1 fragment of SARS-CoV Urbani Spike (S) protein, with an N terminal C5 signal sequence and a C-terminal human IgG Fc [14], was used as a template in site directed mutagenesis PCR using QuikChange Lightning Site-Directed Mutagenesis Kit (Stratagene) to generate Sin845, GZ-C, GDO1, and GZ0402 mutants. The same procedure and primers were used for the generation of the full length S protein mutant constructs using the pcDNA3.1- S, coding for the full length SARS-CoV S protein with a C-terminal (C9) tag derived from human rhodopsin protein, as a template.

### Construction of S-ectodomain, S2, HR1 and HR2 Domains Expression Plasmids

The pcDNA3.1 S encoding the full length S protein of SARS-CoV was used as a template in a PCR reaction to amplify the S-ectodomain (residues 12-1184), the S2 (residues 700-1184), the HR1 (residues 901-1040), and the HR2 (residues 1141-1184) domains. All the forward primers were designed with a 5′ NheI site while the reverse primers were designed with a 5′ BamHI site. The PCR products were then cloned in frame into the C-terminus IgG tag mammalian expression vector [14].

### Expression and Purification of SARS-CoV12-510 S1-IgG Urbani and Mutant Proteins as well as S-ectodomain, S1, S2, HR1 and HR2 Domain Proteins

The plasmids coding for 12-510 S1-IgG proteins as well as the S protein truncations (S-ectodomain, S2, HR1 and HR2 domains) were used to transfect 293FT cells by calcium phosphate transfection method and the proteins were purified using protein A agarose beads as described previously [19]. The purified proteins were concentrated through Centricon filters (Millipore, Bedford, MA) then detected by Coomassie blue staining (Bio-Rad, Hercules, CA) following separation on a 4–15% SDS/PAGE gel. The expression was further confirmed by western blot using polyclonal goat anti-human IgG Fc HRP antibody (Promega).

### Purification of the Non S1 Binding and Neutralizing Human mAbs

Hybridomas of 56 neutralizing non S1 binding HmAbs were cloned by limiting dilution and the clones were cultured in DMEM medium supplemented with 10% Fetal clone (Hyclone laboratories, Logan, Utah) to produce large quantities of HmAbs. The HmAbs were purified using protein-A agarose beads. Thirty nine HmAbs were successfully purified and the Ab production was confirmed by 4–15% SDS/PAGE followed by Coomassie blue staining. The antibody concentration was measured at 280 nm using the Biomate3S UV-Visible Spectrophotometer (ThermoScientific). All HmAbs were diluted to a final concentration of 50 µg/ml.

### Enzyme Linked Immunosorbent Assay (ELISA)

Medisorp ELISA plates (Nunc, Roskilde, Denmark) were coated with 100 ng/well of 12-510 S1-IgG Urbani protein as well as mutant proteins (Sin845, GZ-C, GD01 and GZ0402) overnight at 4°C. The binding of the 18 HmAbs were tested by ELISA as described previously using antihuman IgG2 HRP mouse monoclonal antibody as the secondary antibody (SouthernBiotech, Birmingham, AL) [19]. The same procedure was followed for testing the binding of 39 non S1 neutralizing HmAbs against S protein ectodomain, S2, HR1, HR2 and S1 domain proteins.

### Production of Urbani and Different Mutant Pseudotyped Viruses

Pseudotyped viruses were generated by co-transfection of 293FT producer cells (grown in DMEM with 10% FBS) with pHIV-GFP-luc expression vector, pgagpol HIV vector, pHIV-Rev and pHIV-TAT [31], along with the pcDNA3.1-S coding for the SARS-CoV S protein using calcium phosphate transfection according to the previously described protocol [19]. For the production of HIV/ΔE, only HIV vectors were transfected into the cells. The media were changed the following morning and the supernatants were collected 24 and 48 hrs later and pooled. The pseudotyped viruses were concentrated through a 20% sucrose cushion at 41,000 rpm using Beckman Ultracentrifuge. The incorporation of the S proteins in the virus particles was confirmed by western blot using 1D4 anti-rhodopsin mouse monoclonal antibody (Santa Cruz Biotechnology, Santa Cruz, CA), while the virus p24Ag content was confirmed by mouse anti-HIV1 p24 monoclonal antibody (Santa Cruz Biotechnology, Santa Cruz, CA).

### In vitro Pseudotyped Virus Neutralization Assay

Entry inhibition was performed by pre-incubating Urbani and mutant pseudoviruses, [equivalent to 10 nanograms of p24 Ag, quantified by HIV-1 p24 ELISA kit (Express Biotech International, MD)], with purified mAbs individually or in combinations at 37°C for 1 hr. The pseudovirus/mAb mixture or pseudovirus alone was added to the target 293/ACE2 stable cell line plated at a density of 60,000 cells/well in 12 well plate, and incubated overnight at 37°C and the medium was replaced the following morning. Forty eight hours later, the cells were lysed and luciferase expression was determined using luciferase assay kit (Promega, WI) according to the manufacturer’s instructions. The rabbit immune serum was used as a positive control for entry inhibition. The percentage entry inhibition was calculated using the following equation:
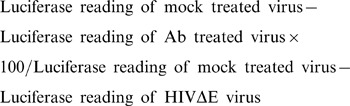
The antibody mediated inhibitions of different mutant pseudoviruses were then normalized to HIV/Urbani-S inhibitions.

## Results

### Expression of SARS-CoV 12-510 S1 IgG Urbani and Mutant Proteins

The SARS-CoV S protein consists of S1 domain in which RBD contains the major neutralizing epitopes, and S2 domain which consists mainly of HR1 and HR2 domains (Fig. S1A). To identify broadly neutralizing HmAbs, we wanted to test our HmAbs against a relatively large panel of variants. We aligned the RBD amino acid sequences available from 94 SARS-CoV late clinical isolates and found mutations in the RBD region of only four clinical isolates relative to the Urbani RBD sequence (Fig. S1B). The clinical isolates with the identified RBD mutations are named Sin845, GZ-C, GD01 and GZ0402 (GenBank accession number: AY559093.1, AY394979.1, AY278489.2 and AY613947.1 respectively). We inserted the identified mutations within the RBD by site directed mutagenesis into the Urbani 12-510 S1 sequence that is fused to human IgG1 Fc tag at the C-terminus [14]. The Urbani and the mutated 12-510 S1-IgG proteins were expressed in 293FT cells, purified and analyzed by SDS/PAGE (Fig. S2A) followed by western blot (Fig. S2B).

### S1 Proteins Containing RBD Sequences of Sin845, GD01, and GZ0402 Isolates Show Low Binding to S1 Specific Neutralizing HmAbs, While that of GZ-C Isolate Shows Higher Binding

**Figure 1:**
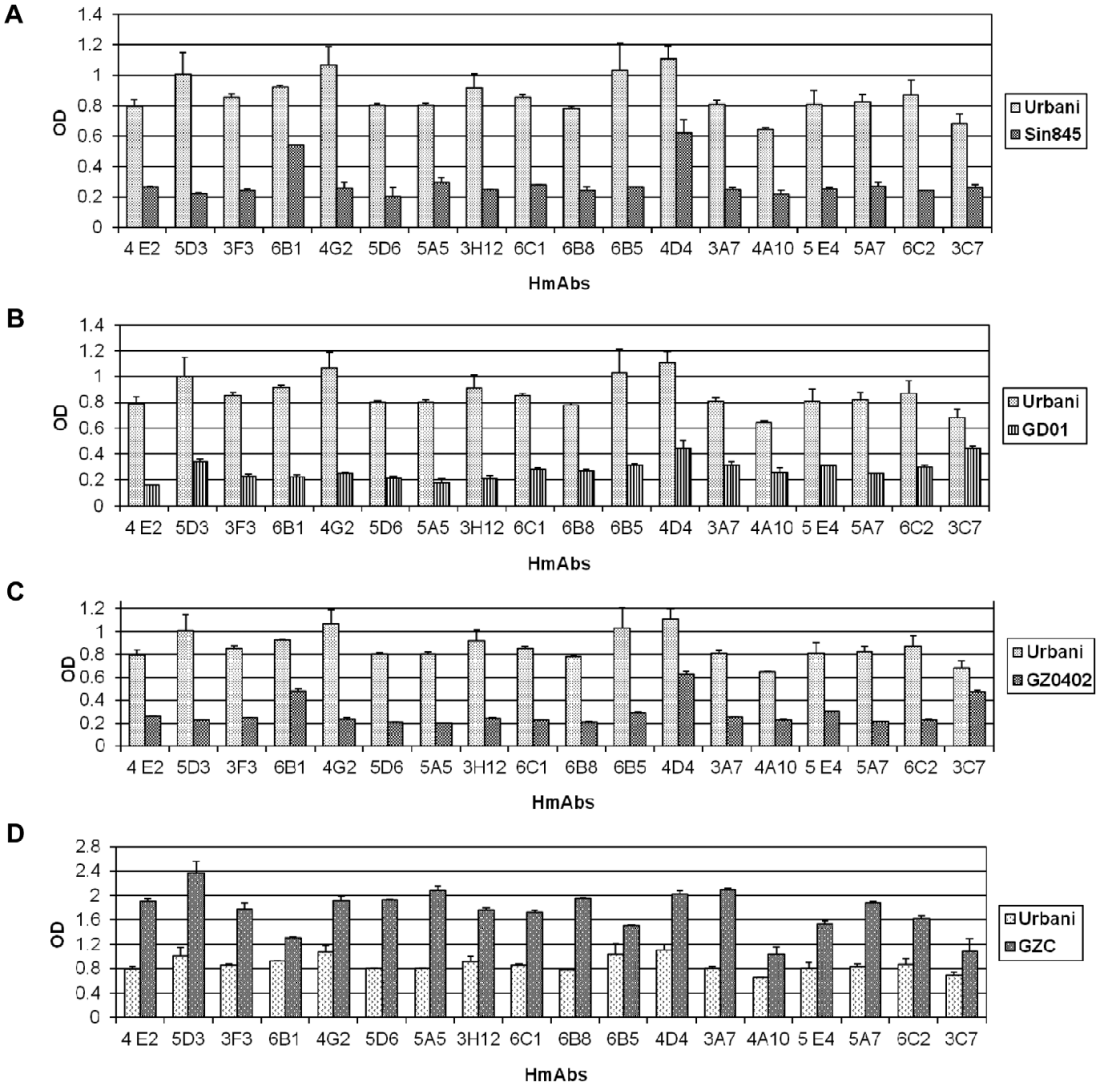
Reactivity of the18 Neutralizing HmAbs with SARS CoV 12-510-S1 proteins. Medisorp ELISA plates were coated with 100 ng/well of Urbani and RBD mutant 12-510S1-IgG proteins and 2.5 µg/ml of each HmAb was used as the primary antibody. Anti-human IgG2 HRP mouse monoclonal antibody was used as secondary antibody. OD was measured at 450 nm. Error bars represent SD of a representative experiment performed in triplicates. (A) Urbani versus Sin845 mutant. (B) Urbani versus GD01 mutant. (C) Urbani versus GZ0402 mutant. (D) Urbani versus GZ-C mutant.

Relative binding of HmAbs to different S1 proteins at different concentrations of antibodies was determined. The binding at the highest concentration used (2.5 µg/ml) is shown. Interestingly, the Sin845-S1 protein failed to react with 16/18 HmAbs (OD ∼ 0.2) when compared to the control OD of ∼0.156. However, HmAbs 4D4 and 6B1 showed about 50% binding to Sin845 S1 protein relative to their binding to Urbani S1 protein (Fig. 1A). The GD01-S1 protein showed a diminished binding to 16/18 HmAbs and binding of about 40% and 60% to 4D4 and 3C7 HmAbs respectively (Fig. 1B). The GZ0402-S1 protein showed minimal binding to 15/18 HmAbs, and 57%, 52% and 69% binding to HmAbs 4D4, 6B1 and 3C7 respectively (Fig. 1C). Surprisingly, the GZ-C S1 protein showed an increased binding to all 18 HmAbs (Fig. 1D).

The diminished binding to the Sin845, GD01 and GZ0402 mutants was further confirmed by the minimal to no binding of the HmAbs 5A5, 5D6 and 4G2, even when the wells were coated with an excessive amount of mutant S1 proteins (i.e. 600 ng) relative to their significant binding to only 100 ngs of the Urbani-S1 protein (data not shown).

**Figure 2:**
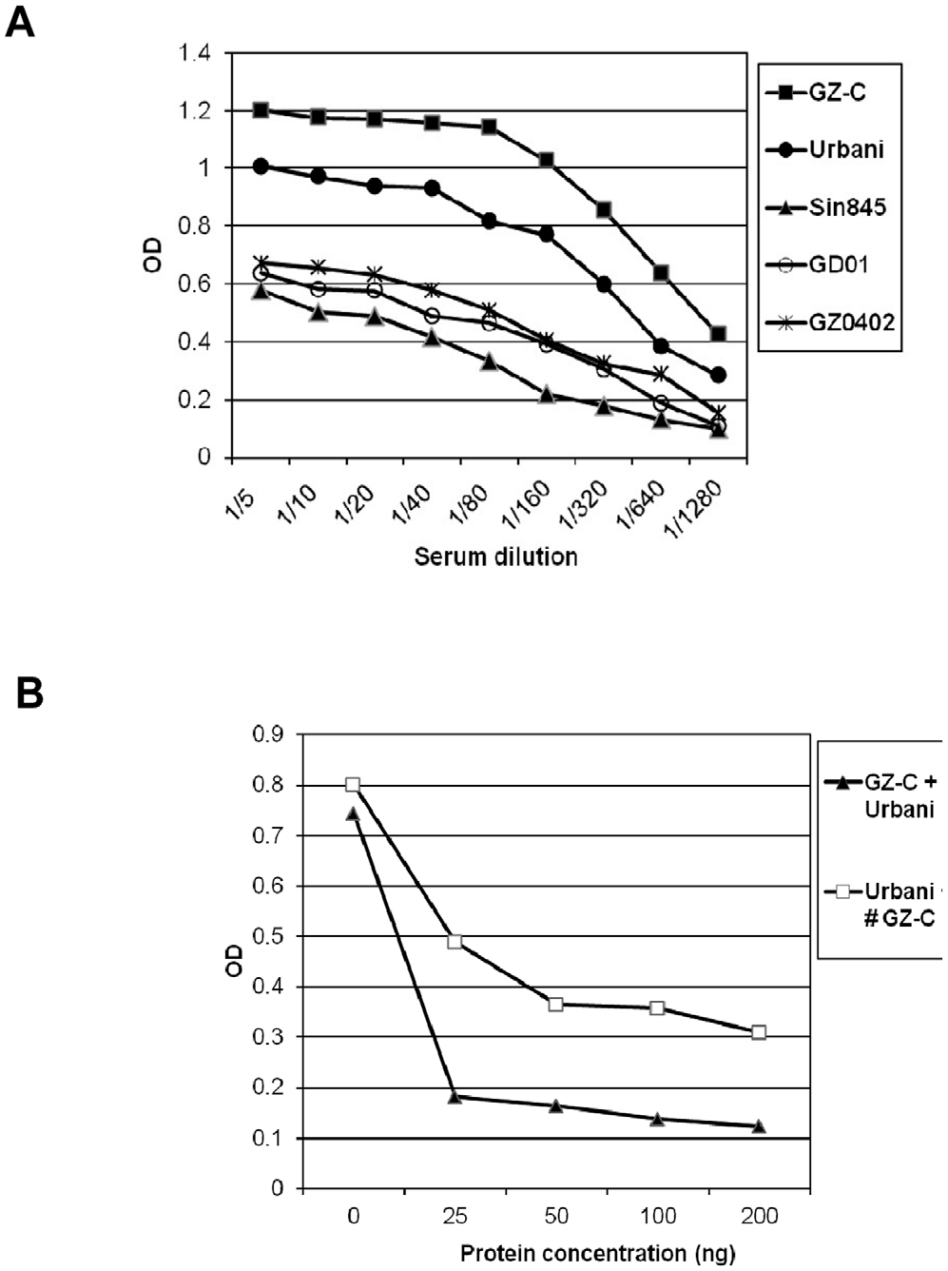
Reactivity of Urbani SARS-CoV-S protein antibodies with Urbani S1 protein and mutant S1 proteins. (A) Different dilutions of a rabbit anti-Urbani SARS-CoV-S protein immune serum were tested in an ELISA against Urbani as well as mutant S1-IgG proteins. Anti-rabbit donkey polyclonal HRP antibody was used as the secondary antibody. (B) Competitive ELISA assay: Different protein concentrations of Urbani or GZ-C proteins were pre-incubated with 5A7 antibody then the protein/Ab mixtures were tested for binding to the other protein by ELISA. OD was measured at 450 nm.

The validity of these findings was confirmed when we found that an anti-SARS-CoV-S Urbani polyclonal serum showed strong reactivity (OD ∼ 0.4) against the GZ-C-S1 mutant even at a high dilution (1/1280) while it showed much lower binding to Sin845-S1, GD01-S1 and GZ0402-S1 proteins relative to its binding to the Urbani-S1 protein (Fig. 2A). Enhanced binding to GZ-C-S1 protein was further validated when we found that as little as 25 ng of the GZ-C protein could block HmAb 5A7 binding to the Urbani-S1 protein while as much as 200 ng of Urbani protein was significantly less efficient in blocking the HmAb 5A7 binding to GZ-C-S1 protein (Fig. 2B).

### S Proteins Containing RBD Sequences of Sin845, GD01, GZ0402 and GZ-C Isolates do not Affect Pseudovirus Entry

We prepared pseudoviruses expressing S proteins containing RBD sequences of Sin845, GD01, GZ0402 and GZ-C isolates to serve as “RBD surrogates” for those clinical isolates. The S protein and the HIV p24 Ag incorporation into the viral particles were confirmed by western blot (Fig. S3A). In HIV/ΔE, as expected, no surface glycoprotein was detected. Pseudoviruses expressing the mutant S proteins entered 293 cells, stably expressing ACE2, with equal efficiency when compared to the HIV/S positive control (Fig. S3B).

### Pseudoviruses Containing S Proteins with RBD Sequences of Sin845, GD01 and GZ0402 Isolates Escape Neutralization While GZ-C Shows Enhanced Neutralization by S1 Specific HmAbs

**Figure 3:**
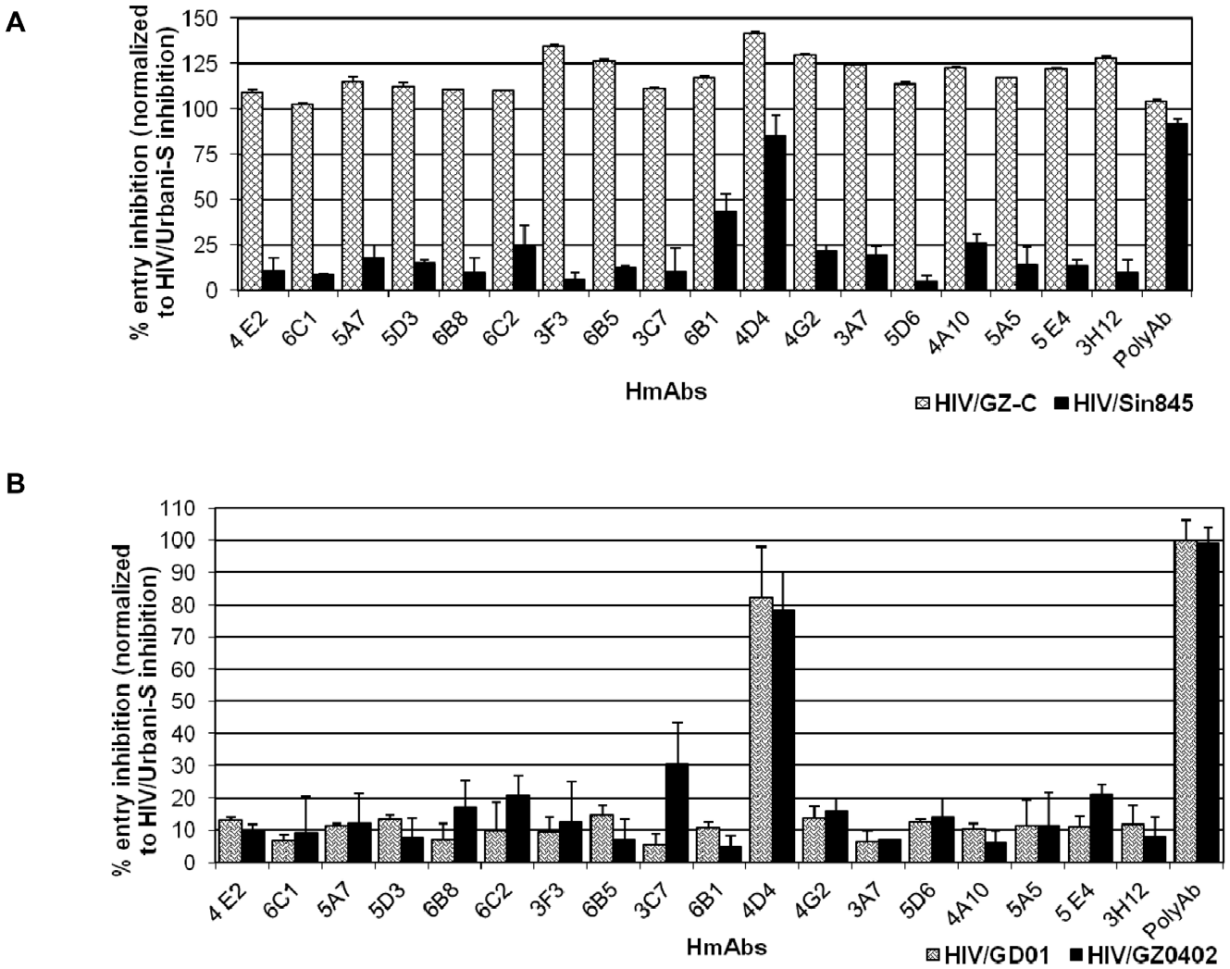
*In vitro* pseudovirus neutralization assay. Eighteen neutralizing HmAbs were tested against different mutant as well as Urbani pseudoviruses. Pseudoviruses equivalent to 10 ng of HIVp24 were incubated for 1 hr with 25 µg/ml of each of the HmAbs at 37°C. The virus/Ab mixtures were then added to 293/ACE2 stable cell line. Seventy two hours later, the virus entry was determined by luciferase expression. The percentage entry inhibitions obtained with Abs were calculated and normalized to HIV/Urbani-S inhibitions (A) HIV/GZ-C and HIV/Sin845 inhibitions (B) HIV/GZ0402 and HIV/GD01 inhibitions. Polyclonal rabbit immune serum (PolyAb) was used as a positive control. Error bars represent SD of a representative experiment performed in triplicates.

Consistent with the binding data shown above, entry inhibition of Sin845-S, GD01-S and GZ0402-S pseudoviruses ranged from 10–45%, except for the HmAb, 4D4, which showed 78–85% inhibition, relative to that seen with Urbani-S pseudovirus by the corresponding antibodies (Fig. 3A, 3B). In contrast, these antibodies showed more efficient inhibition of GZ-C mutant (Fig. 3A). The HmAbs did not show significant inhibition of VSV-G pseudotyped virus which ensures the specificity of the HmAbs (data not shown).

### Differential Reactivity of Non-S1 Binding HmAbs with S Ectodomain, S2 Domain, HR1 and HR2 Regions Suggest Multiple Mechanisms of Virus Neutralization

The recombinant S protein ectodomain, S2 domain, HR1 and HR2 proteins were expressed in 293FT cells and purified using protein-A agarose beads (Fig. S4). Thirty nine non-S1 binding but Urbani strain S-ectodomain binding and neutralizing HmAbs [19], were successfully purified and tested for binding to different regions of the S protein, including S1 domain as a negative control and full-length S-ectodomain as a positive control. OD which is 3x negative control (control OD ∼ 0.13) was considered positive. Twenty two HmAbs bound to S2 domain out of which nine and thirteen bound specifically to the HR1 and the HR2 regions respectively (Table S1). Interestingly, seventeen HmAbs bound to S-ectodomain but failed to bind to HR1 and HR2 regions of the S2 domain.

Inhibition of different pseudoviruses entry by HR1 and HR2 binding HmAbs ranged from 60 to 110% of the Urbani-S pseudovirus inhibition at an antibody concentration of 25 µg/ml (Table 1). In contrast, the S-ectodomain binding HmAbs were less effective and showed entry inhibition ranging from 10–45% of Urbani-S inhibition, except for the HmAb 4G10 which showed ∼76% neutralization of Sin845-S virus, and the HmAbs 3F1 and 2G11 which showed 92% and 98.4% neutralization of the GZ-C-S virus (Table 1). Collectively, the above results showed that the HR1 and HR2 binding HmAbs are more effective in inhibiting the entry of the RBD surrogate clinical isolates. Those HmAbs did not inhibit the entry of VSV-G pseudotyped virus (data not shown).

**Table 1:** HmAbs to HR1 and HR2 can efficiently neutralize surrogate clinical isolates.

Virus	Sin845	GZ-C	GD01	GZ0402	
Ab	Percentage entry inhibition (normalized to HIV/Urbani-S inhibition)				BR^a^
1F1	11.5	24	20.3	16.4	**S-ect** b
3F1	14.4	92	12.8	28.6	**S-ect** b
4 E11	20.4	16.5	18.8	14.8	**S-ect** b
6C5	8.5	29.5	22.4	16.8	**S-ect** b
4G10	76.3	13	12	18	**S-ect** b
3F9	16.7	12.2	17	32.5	**S-ect** b
6D8	10.3	20	16.4	11.8	**S-ect** b
2C6	14.8	15	22.8	21.2	**S-ect** b
2G11	26	98.4	23	21	**S-ect** b
1D11	10	30	13.9	26	**S-ect** b
4 E6	20.2	27.7	20.2	15	**S-ect** b
1C1	28	35.5	22	43.3	**S-ect** b
2B9	16	21.2	21.5	23	**S-ect** b
2 E11	24	31.4	24	30.3	**S-ect** b
1G12	14	13.6	24.2	14.5	**S-ect** b
6H6	31.7	33.2	15.4	21.5	**S-ect** b
1D5	31	21.5	29.4	33.7	**S-ect** b
1F8	84.7	97.7	76	74.8	**HR1**
4A4	84	91.3	89	73	**HR1**
1D12	87.3	98.3	78	68.7	**HR1**
2A12	73.4	96.7	89.4	91.2	**HR1**
5C3	84	88.2	68	89.3	**HR1**
2B12	82	83.7	85.6	89	**HR1**
6H2	89.4	104.8	88	95	**HR1**
6C9	84	81.4	88	79	**HR1**
4F9	80	82.3	90.7	80.8	**HR1**
5G8	89	84	86.8	92.4	**HR2**
5B10	87.6	95	96.5	92.2	**HR2**
3A11	91.6	109	83.5	100	**HR2**
5E9	81.2	96	96.3	96.6	**HR2**
6H1	78.6	96,5	92.3	83.6	**HR2**
1 E10	83	84.6	74.8	86.6	**HR2**
3H11	85.5	95	75.3	84.5	**HR2**
5B9	102.3	94	97.5	110.7	**HR2**
5D7	81	86.3	92	97.7	**HR2**
2D2	89.3	91	94.8	89.2	**HR2**
3 E10	98.4	113	97	105.7	**HR2**
5G9	81.3	107.4	104.7	99.9	**HR2**
2D6	73.2	89.2	96.6	95	**HR2**
PolyAb^c^	97.8	85.4	92	105.6	

a Likely binding region of antibodies.

b S glycoprotein ectodomain.

c Anti-SARS-S protein polyclonal antibody.

### Combinations of SARS-CoV HmAbs Targeted to Different Regions of the S Glycoprotein More Efficiently Inhibit the Entry of RBD Surrogate Clinical Isolates

**Figure 4:**
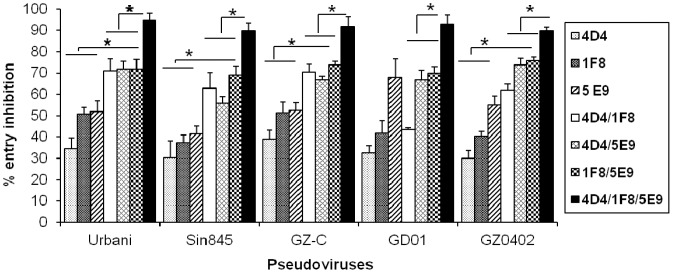
Combinations of HmAbs more efficiently inhibit the entry of SARS-CoV RBD surrogate clinical isolates. Neutralizing HmAbs binding to different regions of S protein 4D4 (S1), 1F8 (HR1), 5E9 (HR2)) were tested for their ability to neutralize pseudoviruses in different combinations as well as individually at a concentration of 6.25 µg/ml each. The virus/Ab mixture was incubated for 1 hr at 37°C then added to 293/ACE2 stable cell line. Seventy two hours later, the virus entry was determined by luciferase expression. The percentage entry inhibitions by individual antibodies as well as combinations of antibodies were calculated. Error bars represent SD of representative experiment performed in triplicates. Statistical analysis was done using Student-t test, significant differences are indicated by asterisks,* p<0.05.

Next, we tested combinations of 4D4 (binds to S1, N-terminal of RBD), 1F8 (binds to HR1) and 5E9 (binds to HR2) HmAbs to see if they can more effectively inhibit viral entry. The combinations of 4D4/1F8, 4D4/5E9 and 1F8/5E9 HmAbs were more effective in blocking Urbani pseudovirus entry compared to the individual antibodies (*p value <0.05*). The same pattern of inhibition was seen with the Sin845-S, GZ-C-S and GZ0402-S pseudoviruses (*p values = 0.005–0.04*). However, these HmAb combinations exhibited similar levels of GD01 pseudovirus blocking as seen with the 1F8 or 5E9 HmAbs when used individually. Maximum inhibition of 90–95% (*p values = 0.003–0.04*) was noted when a combination of 4D4/1F8/5E9 HmAbs was used (Fig. 4). These results indicated that a cocktail of HmAbs targeting different conserved regions of the S protein is likely to be more effective in neutralizing different SARS-CoV clinical isolates than individual antibodies with specificity to those regions.

## Discussion

Therapies that are directed towards RNA viruses, including SARS-CoV, must consider the quasispecies nature of the viral population, the ability of the virus to mutate and recombine in response to host selection pressure [32]. Such changes likely allowed the SARS-CoV to jump from the intermediate hosts to humans and resulted in the 2002–2003 outbreak [33]. Therefore, therapies against SARS-CoV, including passive immunotherapy with HmAbs, must be able to neutralize a wide range of clinical isolates and prevent or minimize generation of escape mutants.

In this study, we found that the anti-S1 HmAbs were unable to bind to the recombinant mutant 12-510 S1 fragments (i.e. Sin845, GD01 and GZ0402) except for the 4D4 antibody, which showed only a decreased binding. All anti-S1 HmAbs showed enhanced binding to the GZ-C-S1 fragment.

The 4D4 HmAb binds to an epitope that resides N-terminal to RBD and neutralizes the SARS-CoV by inhibiting a post-binding step in the viral entry [11], [19]. This HmAb continued to react *albeit* to a lesser extent with surrogate clinical isolates. Moreover, when used in combination with other HmAbs, such as HmAb 3C7, it showed a synergistic effect [11]. Accordingly, our earlier as well as current results highlight the importance of the HmAb 4D4 in neutralizing SARS-CoV mutants and its ability to compliment other HmAbs.

The Identification of S2 domain specific neutralizing HmAbs is consistent with a previous study which showed B-cell responses against the S2 domain in patients who recovered from SARS-CoV infection [34], and other studies which showed that a fragment consisting of amino acids 1055 to 1192 can induce neutralizing antibodies [29], [30]. Therefore, our finding of thirteen neutralizing HmAbs that bind to HR2 domain is consistent with the previous reports on mouse HR2 specific monoclonal antibodies. However those Abs were neither of human origin nor were tested for their ability to neutralize different clinical isolates [27], [35]. Our finding of nine HR1 binding neutralizing HmAbs is novel as there are no reported HR1 specific neutralizing antibodies to date.

We believe that the HmAbs targeted to epitopes within the S1 domain failed to bind and neutralize because of the mutations which most likely disrupted the conformation of the protein and resulted in the loss of expression of specific epitopes. In contrast, S2 domain reactive HmAbs were able to neutralize different RBD surrogate isolates even better than the 4D4 HmAb. Interestingly, analyses of the amino acid sequences of the S protein of 94 SARS-CoV clinical isolates revealed no mutations that are localized to HR1, and only K1163E mutation in the HR2 of six isolates (i.e. SZ3, GZ0402, HSZ-Cb, SZ16, A022, and GZ02), and Q1183R and Q1183K mutations in the HR2 of BJ182-12 and GZ-C isolates respectively. Other isolates were found to be free of any mutations in either HR1 or HR2 domains.

Most of the previously reported HmAbs recognize epitopes within the RBD in which mutations that allow viruses to escape neutralization without loss of infectivity are often found [12], [36]. This is further substantiated in the current study by the loss of neutralization by different RBD binding antibodies due to a single mutation in the S protein. However, HR1 and HR2 regions contain highly conserved neutralization epitopes in which mutations are likely lethal due to their critical role in the membrane fusion required for virus entry. Consequently, as shown by our results, the HR1 and HR2 specific antibodies can neutralize a broad spectrum of SARS-CoV variants with very limited potential, if any, for the emergence of escape mutants, especially when they are used in combination.

Based on results obtained using a combination of mAbs against HBV and RSV, and our previous demonstration of highly efficient neutralization of SARS-CoV using combinations of HmAbs [11], [37], we reasoned that a combination of HmAbs targeting different regions of the S protein would likely confer better protection against different isolates. Combining the S1 binding 4D4 HmAb with either 1F8 (HR1) or 5E9 (HR2) resulted in increased virus neutralization of the mutants Sin845, GZ-C and GZ0402 compared to the individual HmAbs. Failure of the 4D4/1F8, 4D4/5E9 and 1F8/5E9 combinations to increase GD01 pseudovirus inhibition, when compared to the inhibition seen either with 5E9 or 1F8 alone, was likely due to enhanced binding and neutralization of this virus by these HmAbs when used individually. However, a combination comprising of HmAbs 4D4, 1F8 and 5E9 showed further significant increase in virus neutralization compared to each of the individual HmAbs or any of the pairs. These results suggested that the use of a cocktail consisting of HmAbs that can bind to different conserved regions of the S protein may be more desirable for therapeutic use against SARS-CoV infection. This speculation is supported by earlier studies that have shown that it is far more difficult to select for viral variants in the presence of either a polyclonal neutralizing antibody or a cocktail of monoclonal neutralizing antibodies [36], [38]–[40].

Although others have demonstrated the utility of cocktails consisting of mAbs with similar mode of action [12], [36], [41], the present study shows the utility of a cocktail of HmAbs with different specificities and likely with different mechanisms of action for neutralizing a broad spectrum of SARS-CoV clinical isolates. SARS-CoV S protein is a class I fusion protein that contains HR1 and HR2 regions [16], which are highly conserved. Presence of similar structures in many other class I viral fusion proteins [42], point to a common fusion mechanism used by different viruses. Therefore, monoclonal antibodies against such conserved regions might constitute the most effective passive therapy. Our findings are not only relevant to designing a highly effective passive therapy for SARS-CoV but have implications for the development of passive therapy for other viral infections including influenza and HIV [38], [39], [43].

## Supporting Information

Figure S1 **Comparative sequence analysis of the receptor binding domain of spike proteins in SARS-CoV clinical isolates.** (A) Domain structure of the SARS-CoV spike protein (SP; signal peptide, RBM; receptor binding motif, RBD; receptor binding domain, FP; putative fusion peptide, HR1; heptad repeat 1, HR2; heptad repeat 2, TM; transmembrane domain, CP; cytoplasmic domain). (B) Amino acid sequence alignment of aa340-501 within the receptor binding domain (aa318-510) of Urbani SARS-CoV-S protein, Sin845, GZ-C, GD01, and GZ0402 mutant S proteins. Amino acid differences are shown in bold.
(TIF)

Figure S2 **Expression and purification of SARS-CoV S1 proteins (aa 12-510).** 293FT cells were transiently transfected with either Urbani 12-510 S1-IgG expression plasmid or each of the mutant 12-510 S1-IgG plasmids. Recombinant proteins were purified from the supernatants 72 hrs post-transfection using protein-A agarose beads, concentrated and detected by (A) Coomassie blue staining and (B) Western blot using goat polyclonal anti-human IgG antibody.
(TIF)

Figure S3 **Pseudoviruses expressing the spike glycoprotein of clinical isolates entered cells with equal efficiency as HIV/S.** (A) Pseudoviruses, produced by co-transfecting 293FT cells with HIV viral vectors and pcDNA3.1-S encoding the SARS Urbani-S protein or its mutants (i.e. Sin845, GZ-C, GD01 and GZ0402), were concentrated and confirmed for S protein and HIVp24 protein content by western blot. (B) Different pseudoviruses were tested for entry into stable 293/ACE2 cells by measuring the relative luciferase expression (RLU) 72 hrs post-transduction. The HIV/VSVG pseudovirus was used as a positive control and HIV/ΔE as a negative control. Error bars represent SD of representative experiment performed in triplicates.
(TIF)

Figure S4 **Expression and purification of SARS-CoV-S protein domains.** 293FT cells were transfected with the plasmids coding for each of the S protein domains and the proteins were purified from the supernatants 72 hrs post-transfection using protein-A agarose beads, concentrated and detected by Coomassie blue staining of 4–15% SDS/PAGE. (A) S glycoprotein ectodomain, (B) S1 and S2 domain of the S protein, and (C) HR1 and HR2 domains.
(TIF)

Table S1 **Differential reactivity of 39 non-S1 binding SARS-CoV neutralizing HmAbs with Spike protein fragments.**
(DOC)

Information S1 (DOC)
