# Supplementary figures and images for: Human Monoclonal Antibodies against Highly Conserved HR1 and HR2 Domains of the SARS-CoV Spike Protein Are More Broadly Neutralizing
Source: PLoS One. 2012 Nov 21;7(11):e50366. doi: 10.1371/journal.pone.0050366 (PMC3503966; doi:10.1371/journal.pone.0050366)

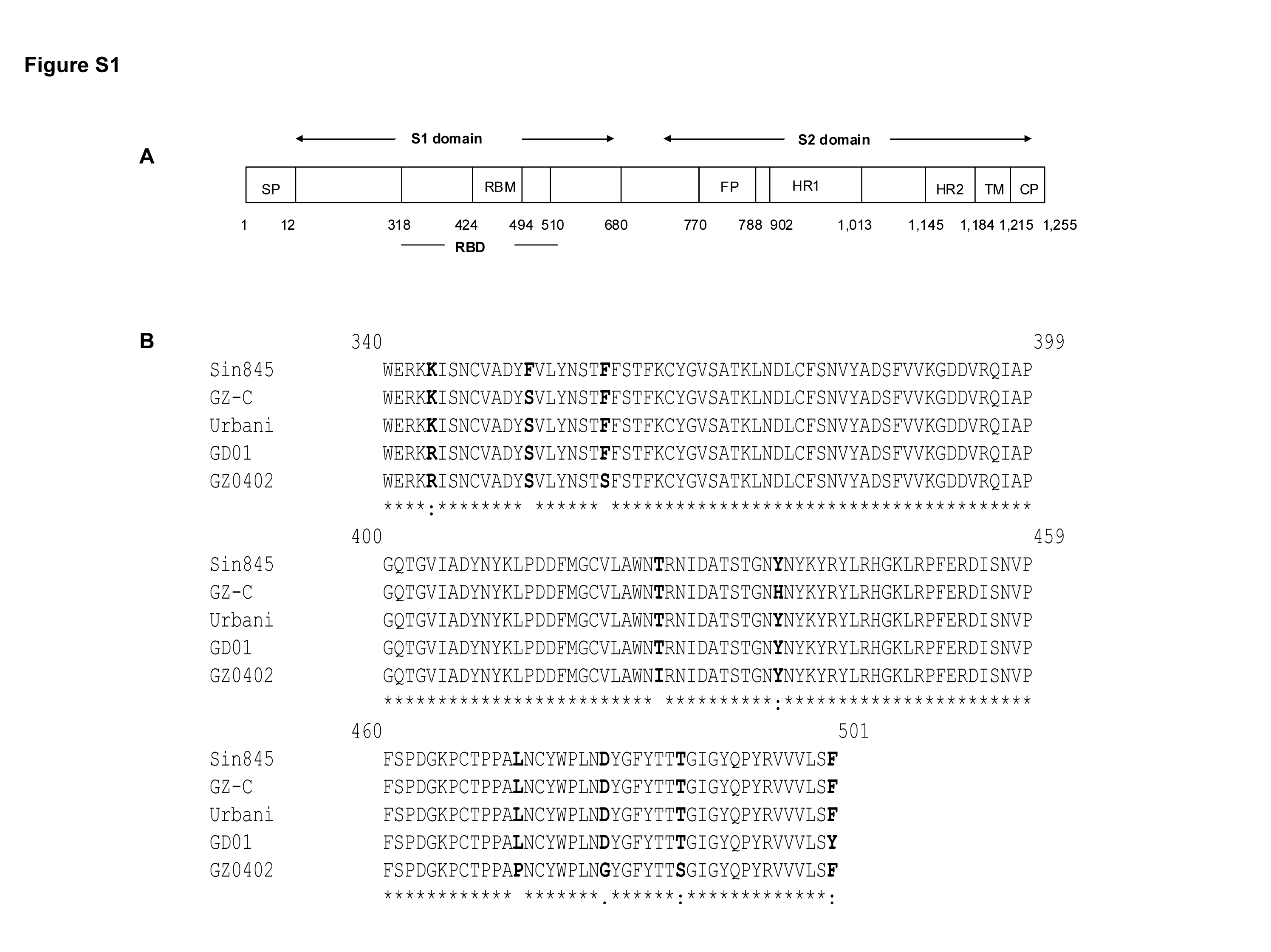

Supplement: Figure S1 — Comparative sequence analysis of the receptor binding domain of spike proteins in SARS-CoV clinical isolates. (A) Domain structure of the SARS-CoV spike protein (SP; signal peptide, RBM; receptor binding motif, RBD; receptor binding domain, FP; putative fusion peptide, HR1; heptad repeat 1, HR2; heptad repeat 2, TM; transmembrane domain, CP; cytoplasmic domain). (B) Amino acid sequence alignment of aa340-501 within the receptor binding domain (aa318-510) of Urbani SARS-CoV-S protein, Sin845, GZ-C, GD01, and GZ0402 mutant S proteins. Amino acid differences are shown in bold. (TIF) [file pone.0050366.s001.tif]

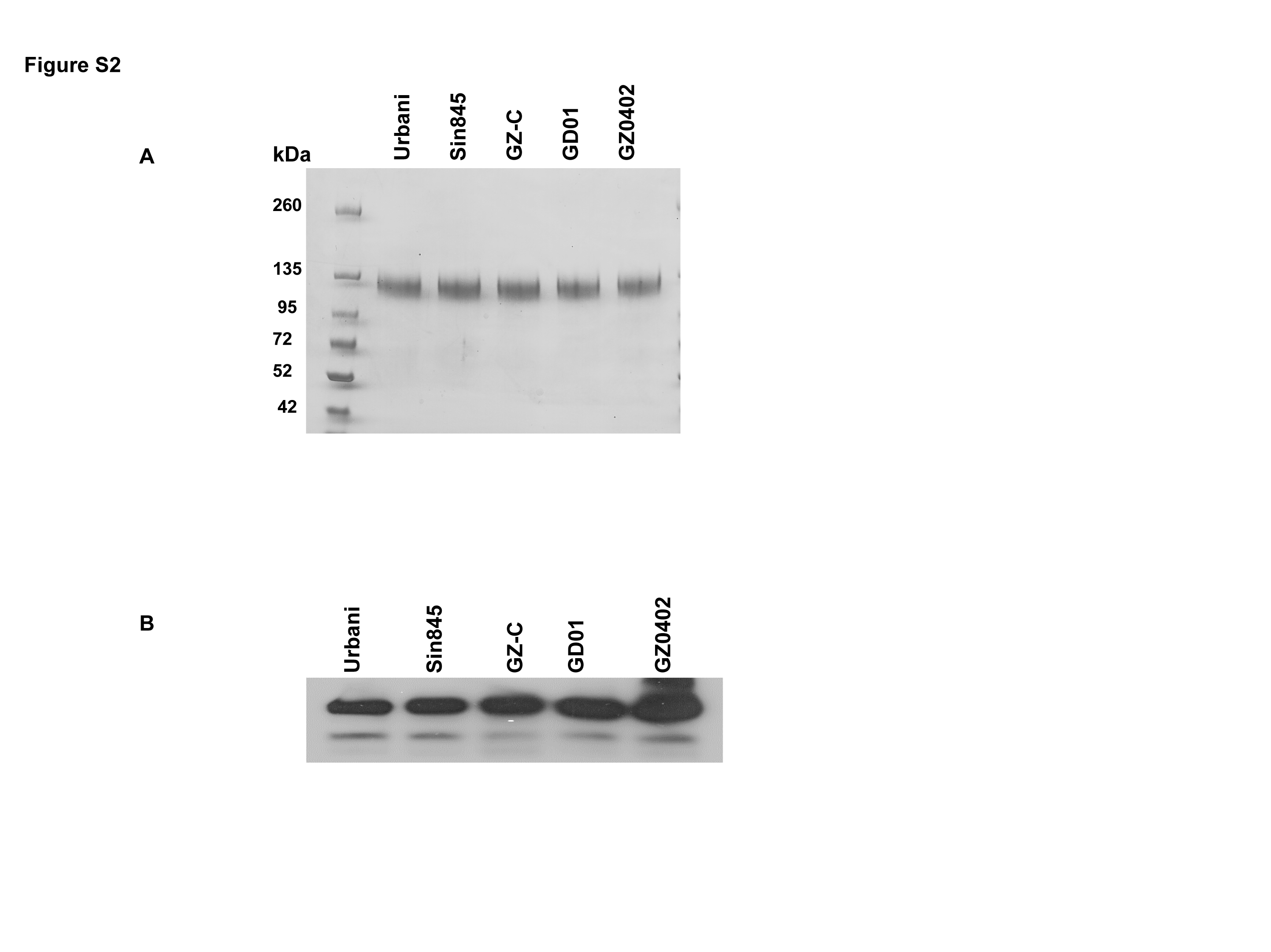

Supplement: Figure S2 — Expression and purification of SARS-CoV S1 proteins (aa 12-510). 293FT cells were transiently transfected with either Urbani 12-510 S1-IgG expression plasmid or each of the mutant 12-510 S1-IgG plasmids. Recombinant proteins were purified from the supernatants 72 hrs post-transfection using protein-A agarose beads, concentrated and detected by (A) Coomassie blue staining and (B) Western blot using goat polyclonal anti-human IgG antibody. (TIF) [file pone.0050366.s002.tif]

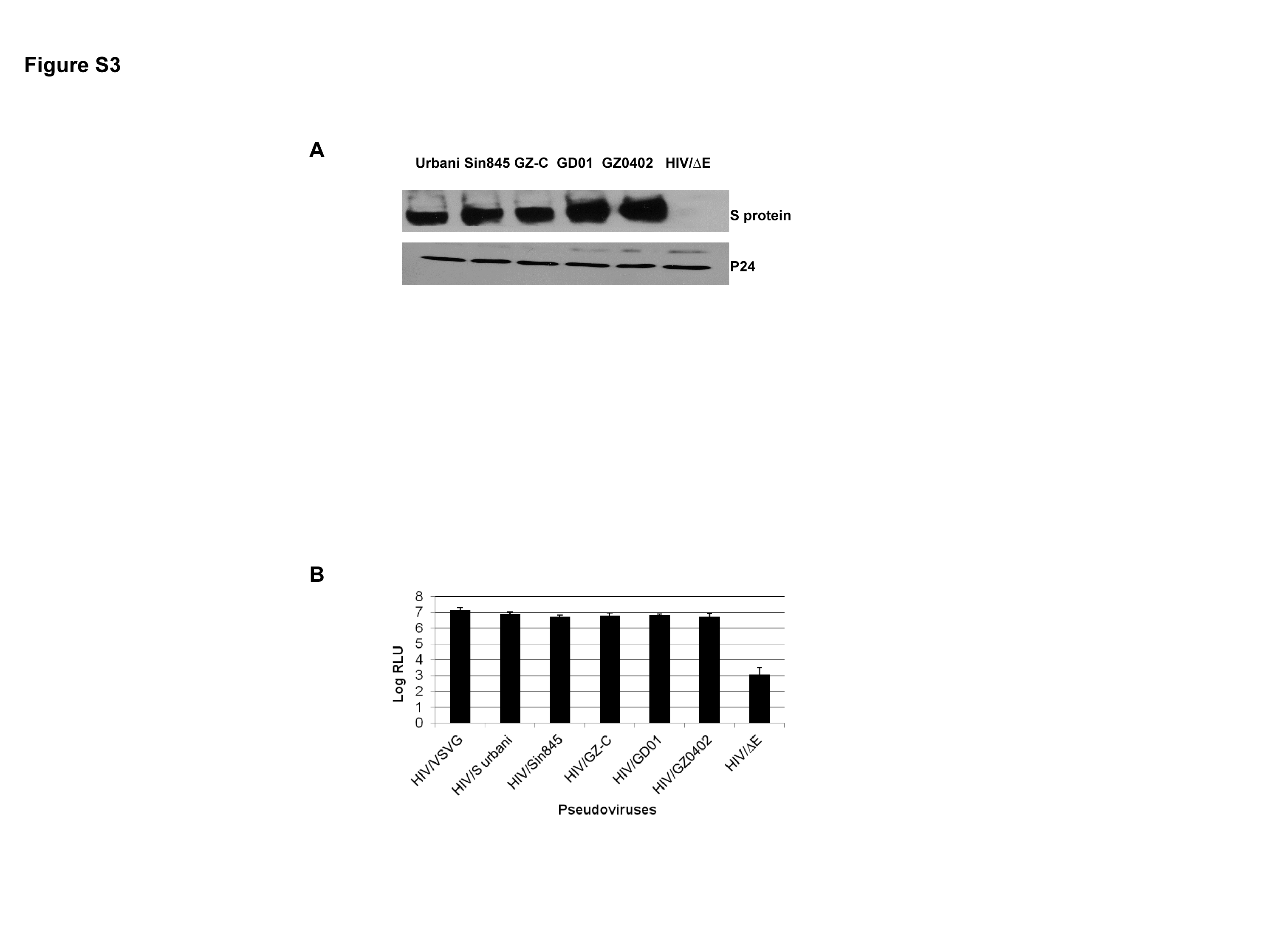

Supplement: Figure S3 — Pseudoviruses expressing the spike glycoprotein of clinical isolates entered cells with equal efficiency as HIV/S. (A) Pseudoviruses, produced by co-transfecting 293FT cells with HIV viral vectors and pcDNA3.1-S encoding the SARS Urbani-S protein or its mutants (i.e. Sin845, GZ-C, GD01 and GZ0402), were concentrated and confirmed for S protein and HIVp24 protein content by western blot. (B) Different pseudoviruses were tested for entry into stable 293/ACE2 cells by measuring the relative luciferase expression (RLU) 72 hrs post-transduction. The HIV/VSVG pseudovirus was used as a positive control and HIV/ΔE as a negative control. Error bars represent SD of representative experiment performed in triplicates. (TIF) [file pone.0050366.s003.tif]

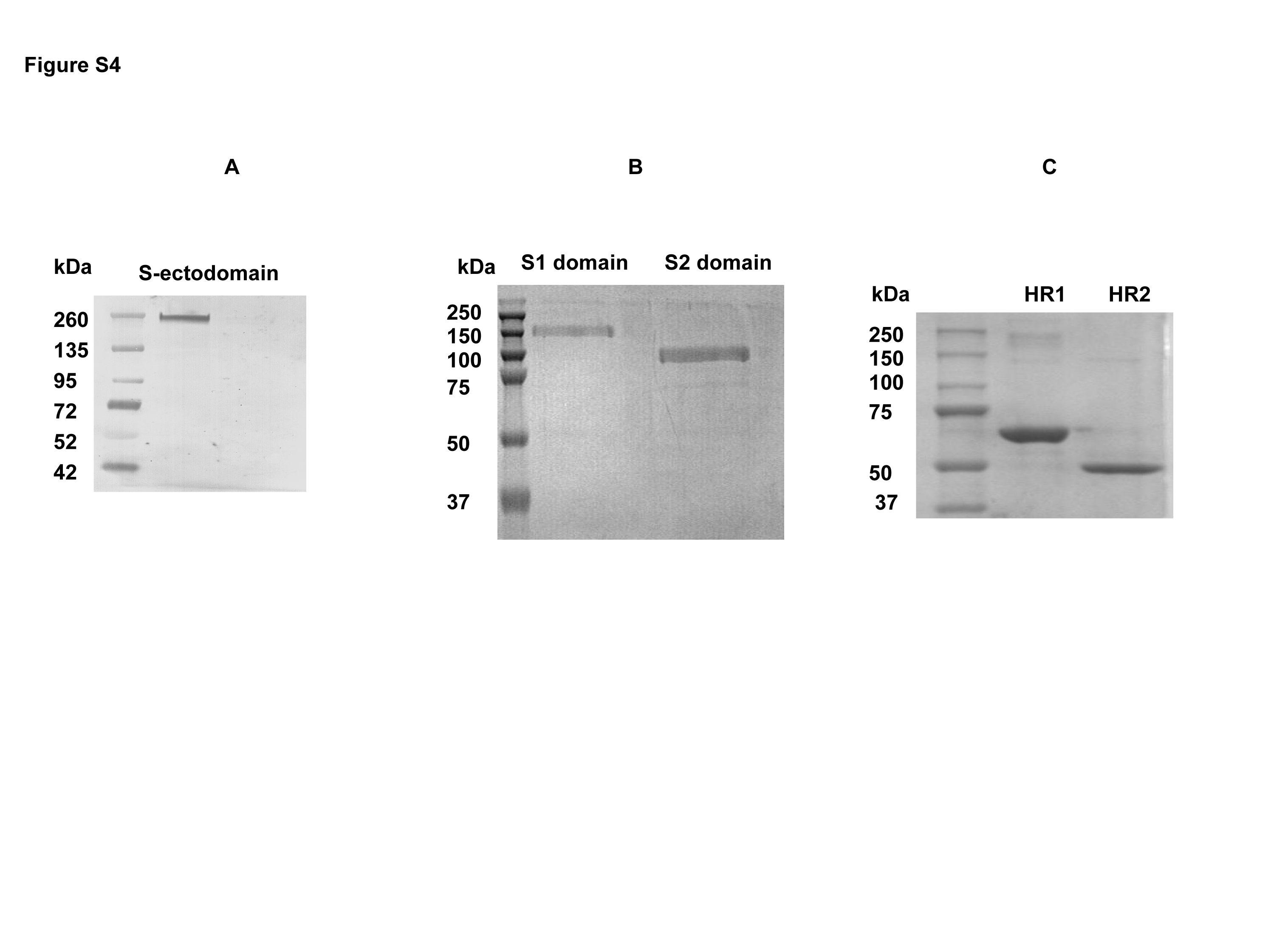

Supplement: Figure S4 — Expression and purification of SARS-CoV-S protein domains. 293FT cells were transfected with the plasmids coding for each of the S protein domains and the proteins were purified from the supernatants 72 hrs post-transfection using protein-A agarose beads, concentrated and detected by Coomassie blue staining of 4–15% SDS/PAGE. (A) S glycoprotein ectodomain, (B) S1 and S2 domain of the S protein, and (C) HR1 and HR2 domains. (TIF) [file pone.0050366.s004.tif]
