# Supplementary material for: Human Monoclonal Antibodies against Highly Conserved HR1 and HR2 Domains of the SARS-CoV Spike Protein Are More Broadly Neutralizing
Source: PLoS One. 2012 Nov 21;7(11):e50366. doi: 10.1371/journal.pone.0050366 (PMC3503966; doi:10.1371/journal.pone.0050366)
Supplement: Table S1 — Differential reactivity of 39 non-S1 binding SARS-CoV neutralizing HmAbs with Spike protein fragments. (DOC) [file pone.0050366.s005.doc]

**Supporting Information Tables:**

| **Table S1. Differential reactivity of 39 non-S1 binding SARS-CoV neutralizing HmAbs with Spike protein fragments.** | | | | | | |
| --- | --- | --- | --- | --- | --- | --- |
| **Ab** | **S-ect.**^a,b^ | **S2^b^** | **HR1^b^** | **HR2^b^** | **S1^b^** | **BR^c^** |
| 1F1 | 0.938 | 0.342 | 0.329 | 0.286 | 0.211 | **S-ect^a^** |
| 3F1 | 0.767 | 0.321 | 0.299 | 0.262 | 0.307 | **S-ect^a^** |
| 4E11 | 0.724 | 0.377 | 0.314 | 0.25 | 0.198 | **S-ect^a^** |
| 6C5 | 0.722 | 0.316 | 0.336 | 0.266 | 0.237 | **S-ect^a^** |
| 4G10 | 0.765 | 0.359 | 0.29 | 0.248 | 0.191 | **S-ect^a^** |
| 3F9 | 0.75 | 0.315 | 0.302 | 0.202 | 0.186 | **S-ect^a^** |
| 6D8 | 0.75 | 0.341 | 0.184 | 0.289 | 0.136 | **S-ect^a^** |
| 2C6 | 0.767 | 0.361 | 0.196 | 0.304 | 0.154 | **S-ect^a^** |
| 2G11 | 0.727 | 0.337 | 0.221 | 0.264 | 0.189 | **S-ect^a^** |
| 1D11 | 0.648 | 0.333 | 0.215 | 0.277 | 0.16 | **S-ect^a^** |
| 4 E6 | 0.699 | 0.346 | 0.217 | 0.259 | 0.179 | **S-ect^a^** |
| 1C1 | 0.651 | 0.349 | 0.225 | 0.252 | 0.153 | **S-ect^a^** |
| 2B9 | 0.613 | 0.266 | 0.208 | 0.251 | 0.204 | **S-ect^a^** |
| 2E11 | 0.697 | 0.319 | 0.21 | 0.311 | 0.168 | **S-ect^a^** |
| 1G12 | 0.642 | 0.276 | 0.227 | 0.345 | 0.239 | **S-ect^a^** |
| 6H6 | 0.659 | 0.356 | 0.239 | 0.332 | 0.207 | **S-ect^a^** |
| 1D5 | 0.69 | 0.334 | 0.274 | 0.313 | 0.226 | **S-ect^a^** |
| 1F8 | 0.758 | 0.529 | 1.036 | 0.323 | 0.203 | **HR1** |
| 4A4 | 0.76 | 0.495 | 0.551 | 0.235 | 0.258 | **HR1** |
| 1D12 | 0.731 | 0.527 | 1.18 | 0.34 | 0.257 | **HR1** |
| 2A12 | 0.869 | 0.557 | 0.753 | 0.299 | 0.256 | **HR1** |
| 5C3 | 0.845 | 0.683 | 1.41 | 0.232 | 0.273 | **HR1** |
| 2B12 | 0.785 | 0.446 | 0.577 | 0.24 | 0.168 | **HR1** |
| 6H2 | 0.624 | 0.403 | 0.519 | 0.28 | 0.189 | **HR1** |
| 6C9 | 0.728 | 0.388 | 0.993 | 0.345 | 0.214 | **HR1** |
| 4F9 | 0.747 | 0.528 | 0.681 | 0.276 | 0.27 | **HR1** |
| 5G8 | 0.746 | 0.533 | 0.353 | 0.703 | 0.297 | **HR2** |
| 5B10 | 0.703 | 0.459 | 0.246 | 0.525 | 0.193 | **HR2** |
| 3A11 | 0.738 | 0.423 | 0.186 | 0.453 | 0.172 | **HR2** |
| 5 E9 | 0.772 | 0.456 | 0.343 | 0.673 | 0.239 | **HR2** |
| 6H1 | 0.744 | 0.548 | 0.252 | 0.571 | 0.282 | **HR2** |
| 1E10 | 0.962 | 0.604 | 0.27 | 0.448 | 0.212 | **HR2** |
| 3H11 | 0.662 | 0.443 | 0.199 | 0.424 | 0.163 | **HR2** |
| 5B9 | 0.7 | 0.434 | 0.35 | 0.626 | 0.161 | **HR2** |
| 5D7 | 0.664 | 0.421 | 0.257 | 0.647 | 0.214 | **HR2** |
| 2D2 | 0.72 | 0.527 | 0.31 | 0.74 | 0.209 | **HR2** |
| 3E10 | 0.859 | 0.601 | 0.307 | 0.52 | 0.333 | **HR2** |
| 5G9 | 0.754 | 0.575 | 0.347 | 0.467 | 0.191 | **HR2** |
| 2D6 | 0.725 | 0.483 | 0.237 | 0.46 | 0.162 | **HR2** |

^a^S glycoprotein ectodomain

^b^Values in the table are average OD of three experiment

^c^Likely binding region
