# Supplementary material for: Human Monoclonal Antibodies against Highly Conserved HR1 and HR2 Domains of the SARS-CoV Spike Protein Are More Broadly Neutralizing
Source: PLoS One. 2012 Nov 21;7(11):e50366. doi: 10.1371/journal.pone.0050366 (PMC3503966; doi:10.1371/journal.pone.0050366)
Supplement: Information S1 — (DOC) [file pone.0050366.s006.doc]

**Supporting Information:**

***Materials and Methods:***

*Construction of expression plasmids for SARS-CoV 12-510 S1-IgG and full length Spike (S) protein mutants.*

To generate the Sin845 mutant (S353F), we used the forward primer 5’GCGTGGCCG ACTACTTCGTGCTGTACAACTCCACC3’ and reverse primer 5’GGTGGAGTTGTACA GCACGAAGTAGTCGGCCACGC3’. For GZ-C mutant (Y436H), we used the forward primer 5’GCCACGAGCACCGGCAACCAT AACTACAAGTATCGC3’ and reverse primer 5’GCGATACTTGTAGTTATGGTTGCCGG TGCTCGTGGC3’, while for GD01 mutant (K344R, F501Y), we used the forward primers 5’GCCTGGGAGCGCAAGAGGATCAG CAACTGCG3’ and 5’GCGTGGTCGTGCTGAGCTACGAGCTCCTGAACGCG3’ and the reverse primers 5’CGCAGTTGCTGATCCTCTTGCGCTCCCAGGC3’, 5’CGCGTTCAG GAGCTCGTAGCTCAGCACGACCA CGC3’ respectively in two independent PCR reactions. The GZ0402 mutant (K344R, F360S, T425I, L472P, D480G and T487S) was generated through five independent PCRs using the following primers: For K344R, the forward primer 5’GCCTGGGAGCGCAAGAGGATCAGCAACTGCG3’ and reverse primer 5’CGCAGTTGCTGATCCTCTTGCGCTCCCAGGC3’. For F360S, we used the forward primer 5’GCTGTACAACTCCACCTCGTTTAGCACCTTCAAGTGC3’ and the reverse primer 5’GCACTTGAAGGTGCTAAACGAGGTGGAGTTGTACAGC3’. For T425I, we used the forward primer 5’GCGTGCTGGCCTGGAACATCCGCAACATCG ACGCC3’ and the reverse primer 5’GGCGTCGATGTTGCGGATGTTCCAGGCCAGCA CC3’. For the L472P mutation, we used the forward primer 5’CCCTGCACGCCGCCCG CCCCGAACTGCTACTGGCCG3’ and the reverse primer 5’CGGCCAGTAGCAGTTCG GGGCGGGCGGCGTGCAGGG3’. Finally for the D480G and T487S mutations, we used the forward primer 5’CCGCTGAACGGCTATGGCTTCTACACTACGTCCGGTAT CGGCTACCAG3’ and the reverse primer 5’CTGGTAGCCGATACCGGACGTAGTGTA GAAGCCATAGCCGTTCAGCGG3’ in a multisite directed mutagenesis PCR using the QuikChange Multi-Site-Directed Mutagenesis Kit (Stratagene). The PCR products were transformed into the Ultracompetent E.coli MC1061/P3 (Invitrogen) and the mutations were confirmed by sequencing (DNA sequencing facility, UIC).

*Construction of S-ectodomain, S2, HR1 and HR2 domains expression plasmids.*

To amplify the S-ectodomain (residues 12-1184), S2 (residues 700-1184) and HR2 (residues 1141-1184) domains, we used the forward primers: 5’TATGCTAGCCAGCGG CAGCGACCTGGACCGC3’, 5’TATGCTAGCCTTCTCCATCAGCATCACGACCGAG3’ and 5’ATCGCTAGCCCACACCAGCCCGGACGTGGACCTG3’ respectively and the reverse primer 5’TATGGATCCCTCTCCTGGAGGTCGATCAGGCTCTCGTTCAG3’. The HR1 domain (residues 901-1040) was amplified using the forward primer 5’ATCG CTAGCCAACCAGAAGCAGATCGCCAACCAG3’ and the reverse primer 5’ATAGGAT CCTCGTGCGGCGCGGCCTGCGGGAAGCT3’. All the forward primers were designed with a 5’ NheI site while the reverse primers were designed with a 5’ BamHI site. The PCR products were then digested with NheI and BamHI restriction enzymes (New England Biolabs, Ipswich, MA) in a step wise manner and cloned into the C-terminus IgG tag mammalian expression vector , which was digested with the same enzymes as well. The ligation was performed using the Quick Ligation Kit (New England Biolabs, Ipswich, MA). The ligation mixtures were transformed into the Ultracompetent E.coli MC1061/P3 (Invitrogen). The inserts were verified by sequencing (DNA Sequencing Facility, UIC)

*Competitive ELISA inhibition assay.*

Different protein concentrations (25, 50, 100 and 200 nanograms) of Urbani or GZ-C protein were pre-incubated with 0.5µg/ml of 5A7 antibody, O/N at 4°C. The protein/Ab mixtures were added to the wells of the ELISA plate previously coated with 100ng of the other protein and incubated for 1hr at RT. The plates were washed and 50μl/well of HRP conjugated mouse anti-human IgG2 monoclonal antibody (Southern Biotechnology, Birmingham, AL) was added and incubated for 1hr at room temperature. Following washing, the antibody binding was detected using 50μl/well of TMB substrate (BD OptEIA, BD Biosciences Pharmigen, San Diego, CA) and the reaction stopped using 25μl of 10% HCl. The absorbance was then read using a microplate reader (Bio-Rad, Hercules, CA) at 450nm.

*ELISA using Rabbit anti-SARS CoV S protein immune serum.*

ELISA plates were coated with 100ngs of the Urbani or mutant SARS-CoV 12-510-IgG recombinant proteins. The rabbit immune serum was used in serial dilutions as the primary antibody and incubated for 1hr at RT with the Urbani and mutant proteins. After washing, 50μl/well of HRP conjugated donkey anti-rabbit polyclonal antibody (GE health care Biosciences, Pittsburgh, PA) was added and incubated for 1hr at room temperature. The rest of the ELISA procedure was as described before.

*Establishment of 293/ACE2 stable cell line.*

Human embryonic kidney cells (293) that have been grown in DMEM/10%FBS were seeded at density of 200,000 cells/well in two 6 well plates. The following day, the medium was changed and one of the two plates was transfected with 0.4µg of human ACE2 expression plasmid using Effectene transfection reagent (Qiagen) according to the manufacturer instructions. The other plate was left as a control. The cells in the transfected and untransfected plate were selected with G418 (Cell gro) 24 hrs post-transfection for 7 days at 2.5mg/ml, 1mg/ml, and 0.8mg/ml (2 wells for each concentration). The stable colonies appearing after 7 days in the transfected plate at 1mg/ml G418 concentration, with complete cell death in the untransfected one, were selected and grown up further more under G418 pressure (0.5mg/ml) and used for the pseudovirus experiments.
